# Altered White Matter and microRNA Expression in a Murine Model Related to Williams Syndrome Suggests That miR-34b/c Affects Brain Development via *Ptpru* and *Dcx* Modulation

**DOI:** 10.3390/cells11010158

**Published:** 2022-01-04

**Authors:** Meitar Grad, Ariel Nir, Gilad Levy, Sari Schokoroy Trangle, Guy Shapira, Noam Shomron, Yaniv Assaf, Boaz Barak

**Affiliations:** 1 Sagol School of Neuroscience, Tel Aviv University, Tel Aviv 6997801, Israel; meitargrad@gmail.com (M.G.); arielnir@mail.tau.ac.il (A.N.); giladlevy@mail.tau.ac.il (G.L.); nshomron@post.tau.ac.il (N.S.); assafyan@tauex.tau.ac.il (Y.A.); 2 Faculty of Social Sciences, School of Psychological Sciences, Tel Aviv University, Tel Aviv 6997801, Israel; sari.trangle@gmail.com; 3 Department of Cell and Developmental Biology, Sackler Faculty of Medicine, Tel Aviv University, Tel Aviv 6997801, Israel; guyspersonal@gmail.com; 4 Edmond J. Safra Center for Bioinformatics, Tel Aviv University, Tel Aviv 6997801, Israel; 5 Faculty of Life Sciences, School of Neurobiology, Biochemistry & Biophysics, Tel Aviv University, Tel Aviv 6997801, Israel

**Keywords:** *Gtf2i*, Williams syndrome, miRNA, *PTPRU*, Rheb1, brain development, miR-34b/c, *Dcx*

## Abstract

Williams syndrome (WS) is a multisystem neurodevelopmental disorder caused by a de novo hemizygous deletion of ~26 genes from chromosome 7q11.23, among them the general transcription factor II-I (*GTF2I*). By studying a novel murine model for the hypersociability phenotype associated with WS, we previously revealed surprising aberrations in myelination and cell differentiation properties in the cortices of mutant mice compared to controls. These mutant mice had selective deletion of *Gtf2i* in the excitatory neurons of the forebrain. Here, we applied diffusion magnetic resonance imaging and fiber tracking, which showed a reduction in the number of streamlines in limbic outputs such as the fimbria/fornix fibers and the stria terminalis, as well as the corpus callosum of these mutant mice compared to controls. Furthermore, we utilized next-generation sequencing (NGS) analysis of cortical small RNAs’ expression (RNA-Seq) levels to identify altered expression of microRNAs (miRNAs), including two from the miR-34 cluster, known to be involved in prominent processes in the developing nervous system. Luciferase reporter assay confirmed the direct binding of miR-34c-5p to the 3’UTR of *PTPRU*—a gene involved in neural development that was elevated in the cortices of mutant mice relative to controls. Moreover, we found an age-dependent variation in the expression levels of doublecortin *(Dcx)*—a verified miR-34 target. Thus, we demonstrate the substantial effect a single gene deletion can exert on miRNA regulation and brain structure, and advance our understanding and, hopefully, treatment of WS.

## 1. Introduction

Williams syndrome (WS) (also known as Williams–Beuren syndrome) is a rare multisystem neurodevelopmental disorder caused de novo by a hemizygous microdeletion of 26–28 genes from the WS chromosomal region (WSCR) on chromosome 7q11.23 [1,2,3]. WS is characterized by particular “elfin” facial features, as well as numerous medical and developmental deficits, including global cognitive impairment [4,5,6,7,8], supravalvular aortic stenosis, diabetes mellitus [9,10], deficient visuospatial skills [11], various musculoskeletal disorders, and many more [2,12,13,14,15,16].

Individuals with WS also exhibit attention deficit hyperactivity disorder (ADHD), obsessive–compulsive disorder (OCD), general anxiety, and specific phobias, but are particularly known for their extreme friendliness (“cocktail party personality”) [3], which oftentimes places them in compromising situations [2,3]. Interestingly, although anxiety symptoms are prominent among most individuals with WS [17,18,19], social anxiety is scarce; individuals with WS exhibit strong inclinations towards social situations, although they lack social inhibitions and many of them are socially isolated [2,3]. The majority of individuals with WS are highly empathic, with relative strength in verbal and language skills [20] (despite their low cognitive capacities), and great interest in music [2,3]. To date, there are no curative therapies for WS, but rather symptomatic treatments that facilitate the burden of medical, developmental, and emotional issues [2].

Among other developmental deficits associated with the disorder [2,16], WS brains are characterized by several abnormal neuroanatomical features, such as smaller brains [21], altered cortical thickness [22,23], and aberrations in gray and white matter (WM) [19,24,25,26,27,28,29,30,31,32,33,34,35,36]. Specifically, the reduced brain volume has mainly been attributed to the decrease in WM in individuals with WS compared to controls [21,34]. Moreover, cortical thickness and sulcal depth have been found to be altered in areas related to social behavior, such as the perisylvian area, orbitofrontal cortex, and insula [21,22,23,24]. In addition, it was previously shown that these vast neuroanatomical changes are already present during childhood [21]—a finding that further emphasizes the developmental component in WS. Regardless, the molecular mechanisms that underlie these characteristic aberrations have not yet been fully identified.

Previous studies [37,38,39,40] have suggested that the increased attraction to social stimuli is somewhat compulsive, and results from abnormal cortical development. For instance, diffusion tensor imaging (DTI) of the prefrontal–amygdala pathways in individuals with WS revealed a reduction in WM integrity compared to controls [30]. Amygdala hyperactivity was previously linked to increased fear and anxiety, more so than to the acquisition of social behavior. Since amygdala activity is regulated by frontal cortex inhibition [3,37,41], the imbalance observed in amygdala activity may be due to frontal cortex aberrations [3,30]. Therefore, it was suggested that the extreme social approach behavior in WS is a product of poor response inhibition due to frontal lobe aberrations [37,38,39]. Congruently, individuals with WS exhibit reduced activity in frontostriatal circuits when tasked with a response-inhibition paradigm [38], and reduced response inhibition has been found to be the strongest indicator of increased social approach behavior in WS children [39].

Additionally, individuals with 7q11.23 duplication (dup7) syndrome—in which the WSCR is duplicated, rather than deleted—exhibit decreased social interaction and poor eye contact relative to typically developed individuals [41,42]. On that account, the notion that the hypersociability phenotype observed in WS is of a genetic basis is reinforced.

Overall, these findings demonstrate that the WSCR is critical for normal behavior and development, and can be affected in a gene dose-dependent manner. WS individuals with atypical deletions that exclude the general transcription factor II-I (*GTF2I*) exhibit reduced social enthusiasm [43] relative to individuals with the typical WS deletion. *GTF2I* encodes the general transcription factor II-I (TFII-I), which influences transcription in numerous biological contexts via binding to other factors involved in transcription, chromatin remodeling, histone modification, and signaling [44]. Additional case studies of individuals with WS with atypical deletions show that the specific deletion of *GTF2I* is linked to the mental retardation and motor deficit phenotypes observed in WS [45,46].

Based on the convergent findings of *Gtf2i*-specific gene-dose-dependent social and anxiety phenotypes in murine models [47,48], which are similar to those observed in the human hemideletion and duplication syndromes [49,50,51], we decided to focus on *Gtf2i* in order to study novel neurobiological mechanisms governing behavioral and neurobiological alterations. Because homozygous *Gtf2i* knockout (KO) in mice is embryonically lethal [52,53,54], past work in this field has not fully exploited the great potential of understanding the interaction between monogenetic developmental aspects in the whole organism, or specific cell types and neural circuits, or how these alter downstream cellular and molecular mechanisms underlying neurodevelopmental and behavioral properties [55].

To overcome these limitations, we previously generated a murine model with a conditional homozygous deletion of *Gtf2i* in forebrain excitatory neurons [56]. The resultant mice, referred to hereafter as NexKO (*Gtf2i*^f/f^:Nex-Cre^+/−^), unexpectedly demonstrated multifaceted myelination aberrations underlying the WS-associated increased sociability and motor deficit behaviors [2,57,58]. Neuronal *Gtf2i* levels, via neuron–oligodendrocyte (OL) interactions, affected the development of proper myelin, leading to axonal conductivity and behavioral deficits that were rescuable by remyelinating or conduction-improving FDA-approved drugs [57,58,59]. Interestingly, myelination deficits were also verified in brain tissue samples from WS individuals, thus establishing our findings of myelination deficits in WS [34,56].

The dramatic transcriptional alterations in multiple mRNAs related to specific myelination and cell differentiation pathways in NexKO mice [56] have led us to explore whether there are master regulators that mediate these changes. The myriad of data supporting the involvement of microRNAs (miRNAs or miRs) in the development of the nervous system [60,61,62,63,64,65,66] encouraged us to explore miRNAs in the context of the neurodevelopmental deficits found in NexKO mice compared to controls. miRNAs are small (~22 nucleotides), endogenous, non-coding RNA molecules that downregulate mRNA expression post-transcriptionally, thus leading to a reduced expression of their protein products [60,67]. mRNA targeting is determined via imperfect base-pairing of the miRNA to the target mRNA’s 3’UTR, while nucleotides 2–8 from the 5’ end of the miRNA (i.e., the “seed” or binding site) achieve a perfect base-pairing with the mRNA’s binding site in its 3’UTR [62,68,69]. This binding induces the mRNA’s degradation, destabilization, or translational inhibition [70]. Currently, there are thought to be more than 2000 reported miRNAs in humans alone [68], which are presumed to regulate ~50% of protein-coding genes in the human genome [69,71]. One miRNA can target hundreds of mRNAs, and in this way influence extensive cellular pathways, acting as a “master regulator” of gene expression [62,68,72]. The largest variety of expressed miRNAs exists in the central nervous system (CNS), thus implicating their importance in CNS regulation [60,61,62,63,64,73]. Moreover, some miRNAs share the same seed sequence, thus creating a cluster of miRNAs that commonly target mRNAs belonging to the same biological pathway or molecular function [60,67], wherein different miRNAs of the same cluster can be differentially expressed, depending on the temporal and spatial context, and can thus fine-tune mRNA expression to yield the optimal protein levels for the specific cellular context [60,67]. Specifically, miRNAs have been shown to play a substantial role in neural progenitor cells’ proliferation, differentiation, and migration [60,63]. Various miRNAs have also been implicated in neurodevelopmental disorders [60], including WS [74], as well as miR-9 and miR-124 in fragile-X syndrome [72], miR-134 in 22q11.2 deletion syndrome [75], and miR-125b [76] and others [77] in autism spectrum disorder (ASD), among many more.

All in all, we conclude that the miRNAs play a key role in regulating the developing nervous system. Here, in order to illuminate molecular and transcriptional mechanisms that are involved in the altered development of the CNS of NexKO mice, we characterized the miRNA regulatory profiles in the cortices of the mutant mice. Furthermore, we characterized neurodevelopmental aspects related to WS, resulting specifically from neuronal deletion of *Gtf2i*.

## 2. Materials and Methods

### 2.1. Mice

*Breeding*: To dissect the function of *Gtf2i* in neurons, *Gtf2i* conditional knockout mice (with homozygous *loxP* sites flanking *Gtf2i*) were crossed with Nex-Cre mice—a Cre line that expresses Cre recombinase selectively in the excitatory neurons of the forebrain, starting around embryonic day (E) 11.5 [56]. Nex-Cre mice are in a C57Bl/6 background, and were previously shown to behave and develop normally [78]. The resulting mice, referred to herein as NexKO (*Gtf2i*^f/f^:Nex-Cre^+/−^), had selective homozygous deletion of *Gtf2i* in the excitatory neurons of the forebrain [56].

*Housing*: Each cage contained 2–4 mice of the same sex, regardless of genotype. Mice were housed at 20–24 °C under a 12 h light–dark cycle (lights on at 07:00, lights off at 19:00), with food and water available ad libitum. All experimental protocols conformed to the guidelines of the Institutional Animal Care and Use Committee of Tel Aviv University, Tel Aviv, Israel. All efforts were made to minimize animal suffering and the number of animals used.

### 2.2. Genotyping

*Tissue and DNA extraction*: Mice were numbered and marked with a specialized animal microtattoo instrument (Fine Scientific Tools, Heidelberg, Germany), and a tissue sample from their tail or ear was taken to determine their genotype. To extract genomic DNA from the tissue, the HotSHOT method [79] was utilized. Each tissue was suspended in 100 μL of alkaline lysis buffer (25 mM NaOH (Bio-Lab Ltd., Jerusalem, Israel) and 0.2 mM EDTA (Sigma-Aldrich, Rehovot, Israel) diluted in DDW) for 30 min at 95 °C while shaking; to terminate the lysis reaction, 100 μL of neutralization buffer (40 mM Tris-HCl (Sigma-Aldrich, Israel) in DDW) was added to the sample, and the mix was cooled at 4 °C for at least 10 min.

To amplify the specific Cre recombinase site, 2 μL from each preparation was added to each PCR reaction. In addition to the sample, each PCR reaction contained 12.5 μL of DreamTaq Green PCR Master Mix (2×) (Thermo-Fisher Scientific, Waltham, MA, USA), 0.5 μL of each Nex-Cre primer (1.5 μL in total; primers were ordered from Hy Laboratories Inc., Rehovot, Israel, and diluted to 10 mM according to the manufacturer’s instructions; for sequences, see Table 1), and 9 μL of DDW. A C1000 Touch thermal cycler (Bio-Rad Laboratories Ltd., Hercules, CA, USA) was used under the following conditions: 95 °C for 4 min, 30 amplification cycles containing 3 temperature steps (denaturing at 94 °C for 30 s, annealing at 55 °C for 30 s, and elongation at 72 °C for 1 min), followed by 7 min at 72 °C and 4 °C until the end.

To determine the mice’s genotypes, 12.5 μL from each PCR product was run on 2% agarose gel (1× TAE (Bio-Lab Ltd., Israel), 2% agarose (Hy Laboratories Inc., Israel), and 3% SERVA DNA Stain Clear G dye (SERVA Electrophoresis GmbH, Heidelberg, Germany), alongside a 100 bp ladder (DM2100 ExcelBand, Smobio Technology, Hsinchu City, Taiwan). Nex-Cre-positive alleles (as expressed in NexKO mice) showed two distinct bands (~770 bp and 525 bp), while Nex-Cre-negative alleles (as expressed in *Gtf2i*^f/f^:Nex-Cre^−/−^ mice; herein referred to as controls) showed one distinct band (~770 bp).

*Sex determination*: To determine the sex of P1 mice, 2 μL from each sample preparation was added to each PCR reaction. In addition to the sample, each PCR reaction contained 12.5 μL of DreamTaq Green PCR Master Mix (2×) (Thermo-Fisher, USA), 0.5 μL of each primer (1.0 μL in total; primers were ordered from Hy Laboratories Inc. (Israel) and diluted to 10 mM according to the manufacturer’s instructions; for sequences, see Table 2), and 9.5 μL of DDW. The C1000 Touch thermal cycler (Bio-Rad Laboratories Ltd., USA) was used under the following conditions: 94 °C for 2 min, 30 amplification cycles containing 3 temperature steps (denaturing at 94 °C for 20 s, annealing at 60 °C for 20 s, and elongation at 72 °C for 30 s), followed by 5 min at 72 °C and 4 °C until the end. The PCR product was run in gel electrophoresis as described above. Males presented two bands in gel (sized 269 and 353 bp), while females presented only one band (269 bp). Sex determination primers and protocols were adapted from Tunster (2017) [80].

### 2.3. Cortex Extraction

Mice were euthanatized via cervical dislocation, and samples from their ears were taken for genotype verification. Following decapitation, once exposed, brains were placed in sterile PBS (Biological Industries, Kibbutz Beit-Haemek, Israel)-containing petri dishes for dissection. Using the OLYMPUS SZ61 stereomicroscope (OLYMPUS, Kyoto, Japan) and clean surgical appliances, cortices were cleaned from surrounding tissues (e.g., basal ganglia, blood vessels) and placed separately in microcentrifuge tubes containing 200 μL of RNAlater solution (Invitrogen by Rhenium, Modi’in, Israel) on ice. Following 24 h of cooling at 4 °C, RNAlater solution was removed, and the cortices were stored at −80 °C until use. All required equipment was sterilized and sprayed with an RNAse inhibitor (RNase-ExitusPlus, Biological Industries, Israel).

### 2.4. RNA Extraction

After thawing on ice, cortices were homogenized in 1 mL of cold TRIzol reagent (Thermo Fisher Scientific, Waltham, MA, USA) using a handheld electric homogenizer (Pro Scientific, Oxford, CT, USA) (for cells undergoing RNA extraction, a strong vortex was suffice for homogenization). After incubation at room temperature (RT) for 5 min, 200 μL of chloroform (Bio-Lab Ltd., Israel) was added to each sample, and tubes were shaken manually for 15 s. Following another incubation at RT for 3 min, tubes were centrifuged for 20 min at 4 °C at full speed (13,800 rpm; Eppendorf Centrifuge 5430R, Eppendorf by Lumitron, Petah Tikva, Israel). Once the mix was separated into three layers, the uppermost RNA-containing clear layer was removed and placed in a fresh tube, to which 1:1 (*v*/*v*) isopropanol (Bio-Lab Ltd., Israel) was added to precipitate the RNA. After briefly shaking the tubes, they were incubated at RT for 5 min, after which they were centrifuged for 15 min at 4 °C at full speed (13,800 rpm). Once the RNA had precipitated, the isopropanol was removed and the pellet was washed twice with 1 mL of 80% ethanol (Sigma-Aldrich, Israel) mixed with DEPC-treated water (Biological Industries, Israel) and centrifuged for 5 min at 4 °C at full speed. After removal of ethanol, the tubes were left to dry for 15–25 min. Once dry, 20–35 μL of DEPC-treated water was added to each tube. Final RNA concentrations were measured using the Thermo Scientific NanoDrop One device (Thermo Fisher Scientific, USA).

### 2.5. Small RNA Sequencing

*RNA quality*: The quality of total RNA extracted from 12 murine cortices was assessed using Agilent’s 4200 TapeStation (Agilent Technologies, Santa Clara, CA, USA), according to the manufacturer’s High Sensitivity Kit protocol. Samples with an RNA integrity number (RIN) of 7.5 or above were considered to be of good quality, and 8 of them (4 per group, NexKO or control) were chosen for small RNA sequencing.

*Sequencing*: A transcriptome library was constructed from all 8 samples using the Illumina TruSeq Small RNA Library Preparation Kit (Illumina, San Diego, CA, USA). Then, sequencing was performed using the Illumina (USA) HiSeq2500 Rapid Run mode with 50 bp single-end configuration, with 15.625 M reads per sample.

*Analysis*: RNA-Seq data were aligned and quantified according to the miRBase mouse assembly (mmu-21), using miR-MaGiC v1.0 [81]. DESeq2 1.24.0 [82] was used to normalize count data and calculate differential expression. miRNAs with FDR-adjusted *p*-values of 0.06 or less were considered to be differentially expressed. Experimentally verified miRNA target genes were obtained through multiMiR 1.10.0 [83] and tested for gene ontology enrichment with clusterProfiler 3.16.1 [84].

### 2.6. Quantitative Reverse Transcription Polymerase Chain Reaction (qRT-PCR)

#### 2.6.1. mRNA Expression

*Reverse transcription*: Extracted total RNA was used as input for mRNA complementary deoxyribonucleic acid (cDNA) synthesis. Reverse transcription (RT) of mRNA was conducted using random primers and the High-Capacity cDNA Reverse Transcription Kit (Thermo Fisher Scientific, USA). The C1000 Touch thermal cycler (Bio-Rad Laboratories, USA) was used under the following conditions: 10 min at 25 °C, 120 min at 37 °C, 5 min at 85 °C, and a final step of 4 °C until the end.

*Real-time quantification*: mRNA expression levels were assessed using the Fast SYBR Green PCR Master Mix (Thermo Fisher Scientific, USA), according to the manufacturer’s instructions, using the Bio-Rad CFX Connect Real-Time PCR Detection System (Bio-Rad Laboratories, USA). Thermal cycler conditions were as follows: 20 s at 95 °C, 40 amplification cycles (3 s at 95 °C to denature, and 30 s at 60 °C to anneal and extend), and a melt curve: 60 °C for 5 s, and an increase of 0.5 °C every 5 s (including a plate read) until reaching 95 °C. Expression values were calculated based on the comparative cycle threshold (Ct) method [85]. Murine mRNA expression levels were normalized to glyceraldehyde 3-phosphate dehydrogenase (*Gapdh*) and human mRNA expression levels were normalized to *β-ACTIN* as endogenous controls (due to their relatively stable expression). mRNA levels are shown as fold change (FC) relative to the control group’s expression levels. Specific primers for the detection of mRNA expression were ordered from Hy Laboratories Ltd. (Israel) and diluted to 10 mM in DEPC-treated water according to the manufacturer’s instructions (see Table 3 and Table 4 for specific sequences).

#### 2.6.2. miRNA Expression

*Reverse transcription*: Extracted total RNA was used as an input for miRNA cDNA synthesis. RT of specific mature miRNAs was conducted using the High-Capacity cDNA Reverse Transcription Kit (Thermo Fisher Scientific, USA), with specific primers from the TaqMan miRNA assays (Thermo Fisher Scientific, USA), according to the manufacturer’s protocol. The C1000 Touch thermal cycler (Bio-Rad Laboratories, Inc., USA) conditions were as follows: 30 min at 16 °C, 30 min at 42 °C, 5 min at 85 °C, and a final step of 4 °C until the end.

*Real-time quantification*: Quantification of mature miRNA expression levels was assessed using the TaqMan Fast Advanced Master Mix (Thermo Fisher Scientific, USA), according to the manufacturer’s instructions, using the Bio-Rad CFX Connect Real-Time PCR Detection System (Bio-Rad Laboratories Ltd., USA). The thermal cycler conditions were as follows: 2 min at 50 °C, 20 s at 95 °C for polymerase activation, and 40 amplification cycles (3 s at 95 °C to denature, and 30 s at 60 °C to anneal and extend). Expression values were calculated based on the comparative Ct method [85]. Mature miRNA levels were normalized to U6 snRNA as an endogenous control (due to its relatively stable expression). miRNA levels are shown as FC relative to the control group’s expression levels. Specific TaqMan assay primers were ordered from Thermo Fisher Scientific (Israel) (see Table 5).

### 2.7. Western Blotting

Brains were dissected from P30 mice and homogenized in solubilization buffer (50 mM HEPES pH = 7.5, 150 mM NaCl, 10% glycerol, 1% Triton x-100, 1 mM EDTA pH = 8, 1 mM EGTA pH = 8, 1.5 mM MgCl_2_, 200 μM Na_3_VO_4_, and protease inhibitor cocktail 1 diluted 1:100 (Merck by Mercury Ltd., Rosh Ha’ayin, Israel)). Equal amounts of protein from each sample were loaded and resolved by SDS–polyacrylamide gel electrophoresis through 12.5% gel. The gel was electrophoretically transferred to a nitrocellulose membrane in transfer buffer (25 mM Tris, 190 mM glycine, and 10% methanol absolute). Membranes were blocked for 45 min in TBST buffer (0.05 M Tris HCl pH = 7.5, 0.15 M NaCl, and 0.1% Tween 20) with 6% skimmed milk, and blotted overnight with rat anti-MBP antibody (Merck MAB386) and rabbit anti-β-Tubulin 4 (Abcam by Zotal Ltd., Tel Aviv. Israel; AB179509) in TBST buffer, followed by a secondary antibody linked to horseradish peroxidase. Immunoreactive bands were detected with the enhanced chemiluminescence reagent.

### 2.8. Cell Culture

Monolayer-adherent HEK-293T cells (transformed human embryonic kidney cells) and SY5Y-SH cells (bone-marrow-derived human neuroblastoma cells) were grown in Dulbecco’s modified Eagle’s medium (DMEM) (Gibco by Thermo Fisher Scientific, USA) supplemented with 10% (*vol*/*vol*) fetal bovine serum (FBS) (Thermo Fisher Scientific, USA) and 0.01% (*vol*/*vol*) pen–strep solution ×10 (Biological Industries, Israel).

Cells were incubated at 37 °C in a 5% CO_2_ atmosphere. Before use, each cell line was confirmed to have no mycoplasma contamination using the EZ-PCR Mycoplasma Detection Kit (Biological Industries, Israel).

Prior to each experiment, the cells were dissociated using trypsin 0.25% EDTA (Thermo Fisher Scientific, Israel), stained with trypan blue (Biological Industries, Israel), and counted using the Countess^TM^ II Automated Cell Counter (Invitrogen at Thermo Fisher Scientific, USA).

### 2.9. Cloning and Site-Directed Mutagenesis

*Polymerase chain reaction (PCR)*: Inserts were amplified from human genomic DNA using Phusion Hot Start Flex DNA Polymerase (New England Biolabs, Ipswich, MA, USA) under the following conditions: 98 °C for 30 s, 30 cycles of 98 °C for 10 s, 55 °C for 30 s, and 72 °C for 30 s for each kb, and final extension of 72 °C for 10 min.

*Restriction*: Double digestion of the backbone and the insert was conducted with two different restriction enzymes (New England Biolabs, USA) and their compatible buffer—CutSmart Buffer 10× (New England Biolabs, USA)—for 1 h at 37 °C, followed by an inactivation step of 20 min at 65 °C, according to the manufacturer’s instructions. The backbone’s 5’-phosphate was then removed using Antarctic phosphatase (New England Biolabs, USA), according to the manufacturer’s instructions. Both the insert (following restriction reactions) and backbone (following restriction and phosphatase reactions) were run on a 0.75% agarose gel stained with ethidium bromide (EtBr) (Hy Laboratories Inc., Israel) to ensure specificity, and then purified using the Wizard SV Gel and PCR Clean-Up System (Promega, Madison, WI, USA) to eliminate excess nucleotides, primers, and previous inserts cloned into the vector.

*Ligation*: The clean restriction products were ligated using T4 DNA ligase and its compatible Ligase Reaction Buffer 10× (New England Biolabs, USA) for 20 h at 16 °C, followed by an inactivation step of 10 min at 65 °C.

*Transformation*: Ligated plasmids were transformed via heat shock (42 °C for 2 min) into DH-5α heat-shock-competent *Escherichia coli (E.coli)* cells (Bio-Lab Ltd., Israel), and then incubated in lysogeny broth (LB) at 37 °C for 1 h while shaking. Then, cells were grown overnight on 0.1% ampicillin LB–agarose plates at 37 °C. Potential colonies were selected to undergo colony PCR for validation of transfection of the desired insert, using the kappa enzyme (Sigma-Aldrich, Israel), under the following conditions: 95 °C for 180 s, 35 cycles of 95 °C for 15 s, 55 °C for 15 s, and 72 °C for 5 s for each kb, and final extension of 72 °C for 10 min. PCR products were run on 0.75% agarose gel stained with EtBr, and colonies that were found to be positive for the desired insert were grown overnight in a 37 °C shaker in 0.1% ampicillin liquid LB. Plasmids were extracted from cells using the HiYield Mini-Prep Kit (RBC bioscience, New Taipei City, Taiwan), and were verified by Sanger sequencing [86] (Zabam at the Faculty of Life Sciences, Tel Aviv University).

*Site-directed mutagenesis*: As a negative control for the luciferase reporter assays (see 3.8), miRNA-binding sites at the 3’UTR-containing psiCHECK^TM^-2 plasmids were mutated using pre-designed primers and the QuikChange Lightning Site-Directed Mutagenesis Kit (Agilent Technologies, USA). Mutagenesis primers were planned according to the QuikChange Primer Design Program (Agilent Technologies, USA), and contained transversion and transition mutations of 4 nucleotides of the miR-34c-5p binding site on the mRNA’s 3’UTR (for primers, see Table 6 and Table 7). Following mutagenesis, the PCR products were enriched via transformation (as discussed above) and verified by Sanger sequencing [87] (Zabam at the Faculty of Life Sciences, Tel Aviv University).

### 2.10. Cloning and Mutagenesis of Human PTPRU 3’UTR

A ~400 bp fragment containing the miR-34c-5p binding sequence in the 3’UTR of the human *PTPRU* was cloned as described above, and inserted into a psiCHECK^TM^-2 plasmid (Promega, USA). For restriction, XhoI and NotI*-*HF restriction enzymes were used (New England Biolabs, USA). Then, the *PTPRU* psiCHECK^TM^-2 was mutated at the miR-34c-5p binding site, as described above. Primers sequenced for fragment amplification, cloning, and mutagenesis can be seen in Table 6.

### 2.11. Cloning and Mutagenesis of Human RHEBL1 3’UTR

A ~250 bp fragment containing the miR-34c-5p binding sequence in the 3’UTR of the human *RHEBL1* was cloned as described above, and inserted into a psiCHECK^TM^-2 plasmid (Promega, USA). For restriction, XhoI and NotI*-*HF restriction enzymes were used (New England Biolabs, USA). DMSO was added to PCR reactions to relieve secondary structures. The *RHEBL1* psiCHECK^TM^-2 was mutated at the miR-34c-5p binding site, as described above. Primers sequenced for fragment amplification, cloning, and mutagenesis can be seen in Table 7.

### 2.12. Cloning of Human pre-miR-34c

A ~150 bp fragment containing the human pre-miR-34c sequence was cloned as described above and inserted into the miRNA expression vector (miRVec) under a strong Cytomegalovirus (CMV) promoter. For restriction, BamHI-HF and EcoRI-HF restriction enzymes were used (New England Biolabs, USA). Since the insert naturally contains an EcoRI restriction site, addition of this restriction site to the 5’ end of the reverse primer used for cloning was unnecessary. Primers sequenced for fragment amplification and cloning can be seen in Table 8.

### 2.13. Plasmid Transfections

HEK-293T or SY5Y-SH cells were seeded in 24-well plates at a concentration of 8 × 10^4^ cells/well. Twenty-four hours later, at ~60% confluence, HEK-293T or SY5Y-SH cells were transfected with 500 ng of plasmid using Lipofectamine 2000 Transfection Reagent (Invitrogen by Thermo Fisher Scientific, USA) and Opti-MEM I 1X (Thermo Fisher Scientific, Israel), according to the manufacturer’s instructions. Transfection efficiencies were determined via RT-qPCR.

### 2.14. Dual Luciferase Assay

As reviewed in Section 3.2, fragments of ∼250–400 bp of *PTPRU* or *RhebL1* 3′UTR spanning the miRNA-binding sites were cloned downstream of the Renilla luciferase reporter of the psiCHECK^TM^-2 plasmid, which also contains a firefly luciferase reporter (used as a control). As negative controls, the miRNA-binding sites were mutated using the QuikChange Lightning Site-Directed Mutagenesis Kit (Agilent Technologies, USA).

For the luciferase assays, HEK-293T cells were transfected using Lipofectamine 2000 Transfection Reagent with 5 ng of psiCHECK-2 plasmid containing the desired 3′UTR, with or without site-directed mutations, and 485 ng of miRVec containing the pre-miRNA-34c insert, or no miRVec at all. At 72 h post-transfection, firefly and Renilla luciferase activities were measured using the Dual Luciferase Reporter Assay System (Promega, USA) and the LUMIstar Omega Luminometer (BMG LabTech, Ortenberg, Germany) (courtesy of Professor Carmit Levy), according to Promega’s instructions.

### 2.15. Diffusion MRI, Fiber Tracking, and Analysis

MRI scanning was performed using a 7 T MRI scanner (Bruker, Billerica, MA, USA) with a 30 cm bore and a gradient strength of up to 400 mT/m. The MRI protocol included diffusion imaging acquisition with a diffusion-weighted spin-echo echo-planar imaging pulse sequence. Acquired volumes were 45 slices, each 0.16 mm thick, with the following parameters: resolution of 0.16 mm × 0.16 mm^2^ (matrix size, 80 × 96), repetition time of 2500 ms, echo time of 18.5 ms, Δ/δ were 10/2.5 ms, 4 echo-planar imaging segments, and 30 non-collinear gradient directions with a single *b*-value shell at 1000 s/mm^2^ and 3 images with a *b*-value of 0 s/mm^2^ (b0 image). The DTI acquisition took 2 h and 12 min.

All diffusion MRI analysis was performed in ExploreDTI [88], and included the following steps:

1. Anisotropic smoothing with a 0.48 mm Gaussian kernel. This procedure de-noises the data and benefits the fiber tracking procedure;
2. Motion and distortion correction to correct for possible motion- and susceptibility-induced artifacts;
3. Transformation into atlas space via nonlinear registration and extraction of atlas space FA and MD per mouse brain;
4. Whole-brain fiber tracking with 0.16 mm × 0.16 mm × 0.16 mm seed voxel resolution; minimal FA and stropping criteria for tracking: FA > 0.05; maximal 30° tracking angle was allowed. Tracking step size: 0.16 mm;
5. Tracking was conducted from two seed regions of interest (ROIs): genu of the corpus callosum (CC) identified on a mid-sagittal plane; and capturing limbic outputs such as the fimbria/fornix fibers and the stria terminalis in the coronal plane, 0.5 mm before the level of the anterior commissure;
6. The reconstructed number of fibers was taken for statistical analysis between groups.

### 2.16. Statistics

Data are presented as the mean ± standard error of the mean (SEM), as calculated by GraphPad Prism 8.4.3. *p*-Values were calculated using Student’s *t*-test, with *p* < 0.05 considered significant (* < 0.05, ** < 0.01, *** < 0.005). Normality of distributions and equality of variances were checked and addressed accordingly by using the appropriate statistical analysis. Outliers were determined via the extreme studentized deviate (ESD) method.

## 3. Results

### 3.1. White Matter Microstructure, Tract Connectivity, and Myelin Deficits in the Brains of 1-Month-Old NexKO Mice

**Figure 1:**
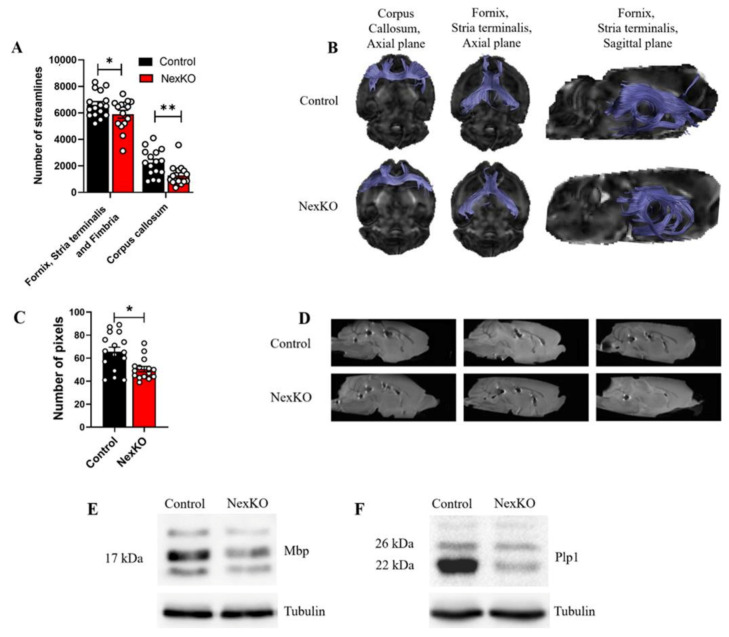
White matter microstructure, tract connectivity, and myelin deficits in the brains of 1-month-old NexKO mice: (**A**) Significantly decreased number of streamlines in the limbic outputs through the fimbria/fornix fibers and the stria terminalis as well as the corpus callosum of NexKO mice compared to controls. (**B**) Brain images overlaid with tractography results for diffusion tensor imaging showing fiber tracking results for control (**upper row**) and NexKO (**lower row**) P30 mice. (**C**) Significantly smaller area of the genu of the corpus callosum of NexKO mice compared to controls. (**D**) Midsagittal brain images from control and NexKO mice, demonstrating the altered anatomical features of the corpus callosum. Western blots of (**E**) MBP isoforms and (**F**) Plp1 expression levels in the cortices of P30 controls and NexKO mice. Data are shown as the mean ± SEM. * *p* < 0.05, ** *p* < 0.01; (**A,C**) two-tailed *t*-test. (**A,C**) *n* = 16 control; *n* = 15 NexKO.

Our previous findings of myelination deficits in NexKO mice as compared to controls prompted us to assess the nature of the WM-related alterations in key WM tracts involved with social cognition and WS-related deficits. To achieve this, we examined the number of streamlines following tractography, and compared P30 NexKO mice to controls. Significantly lower numbers of streamlines were found in limbic outputs of the fimbria/fornix fibers and the stria terminalis (*p* = 0.036) and the CC (*p* = 0.002) of NexKO mice compared to controls (Figure 1A,B).

Additionally, we measured a smaller area of the genu of the corpus callosum (CC) in the midsagittal view in NexKO mice compared to controls, implying thinner and shorter representation of the CC (Figure 1C,D).

To confirm that the altered WM in NexKO mice compared to controls is a result of reduced expression of myelin-related proteins, we measured the expression levels of Mbp and Plp1 in whole cortex, utilizing Western blotting. The expression levels of Mbp (Figure 1E) and Plp1 (Figure 1F) were significantly lower in NexKO mice compared to controls, expanding our knowledge from previous findings on myelination deficits in WS [56].

### 3.2. miRNA and mRNA Expression Is Altered in the Cortices of 1-Month-Old NexKO Mice

**Figure 2:**
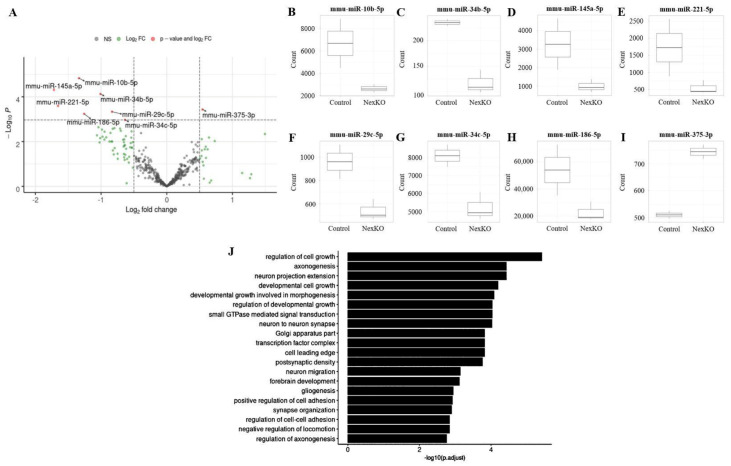
Whole-cortex small RNA sequencing analysis of P30 NexKO mice. Small RNA sequencing analysis of total RNA extracted from the cortices of P30 NexKO mice compared to controls revealed altered miRNA expression. (**A**) Volcano plot representation of differentially expressed miRNAs. Data are shown as the log2 fold change in counts in NexKO mice compared to controls. (**B**–**H**) Seven downregulated and (**I**) one upregulated miRNA in the cortices of NexKO mice compared to controls. Data are shown as medians ± quartiles. *n* = 3 control; *n* = 2 NexKO. (**J**) Gene Ontology analysis of verified targets of the murine miR-34 cluster, presenting biological pathways significantly associated with verified targets of the miR-34 cluster; among them are pathways associated with axonogenesis, forebrain development, gliogenesis, and developmental growth.

The altered neuroanatomy described above, along with the transcriptional alterations we previously found in multiple mRNAs related to specific myelination and cell differentiation pathways in NexKO mice [56], have led us to explore whether miRNAs act as master regulators, potentially responsible for these changes. To achieve this, we isolated small RNA molecules from the whole cortices of 1-month-old NexKO and control mice, and sequenced them to study genome-wide profiling of known miRNAs. Indeed, a bioinformatics analysis of the small RNA sequencing results identified several miRNAs that were differentially regulated in the cortices of 1-month-old NexKO mice compared to controls (Figure 2A–I). Most of these miRNAs were previously linked to brain development, such as miR-10b-5p [87,89], miR-145a-5p [90], miR-221-5p [91], miR-29c-5p [92], miR-186-5p [93], miR-34b-5p [94], and miR-34c-5p [94].

### 3.3. miR-34b/c-5p Expression Levels Are Downregulated in the Cortices of 1-Month-Old NexKO Mice

Among the miRNAs that were found to be significantly downregulated in NexKO mice compared to controls were mmu-miR-34b-5p (Figure 2C) and mmu-miR-34c-5p (Figure 2G; occasionally referred to as miR-34b/c-5p), which belong to the evolutionarily conserved miR-34 family [94,95]. Interestingly, members of the miR-34 cluster were found to be involved in the development of the nervous system (Figure 2J), most notably in cell differentiation and migration [94,96,97,98,99]—processes which we previously found to be disrupted in the cortices of NexKO mice compared to controls [56].

**Figure 3:**
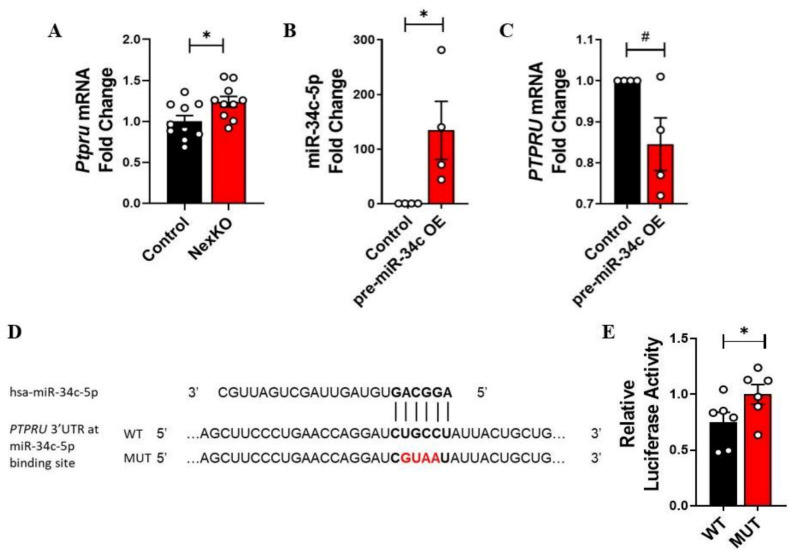
miR-34c-5p directly regulates *PTPRU* mRNA: (**A**) Significantly increased expression levels of *Ptpru* mRNA in the cortices of P30 NexKO mice compared to controls. (**B**) RT-qPCR validation of miR-34c-5p overexpression 72 h post-transfection of hsa-pre-miR-34c in the SH-SY5Y cell line, compared to controls. (**C**) Reduced levels of *PTPRU* mRNA 72 h post-transfection of hsa-pre-miR-34c in SH-SY5Y cells, compared to controls. (**D**) Sequences of the mature hsa-miR-34c-5p and the Renilla/firefly luciferase psiCHECK2 constructs (WT and MUT) under the regulation of *PTPRU* 3’UTR around the hsa-miR-34 binding site; miR-mRNA binding sites are shown in bold; mutated nucleotides are in red. (**E**) Significantly higher luciferase activity levels of the mutated *PTPRU* 3’UTR luciferase construct 72 h post-transfection of hsa-pre-miR-34c in HEK-293T cells, compared to the activity levels of the WT luciferase construct. Data are shown as the mean ± SEM. * *p* < 0.05, # *p* = 0.08; (**A**–**C**) two-tailed *t*-test (**B**,**C**) with Welch’s correction; (**E**) one-tailed *t*-test. (**A**) *n* = 10 per group; (**B**,**C**,**E**) *n* = 6 per group. OE: overexpression.

**Figure 4:**
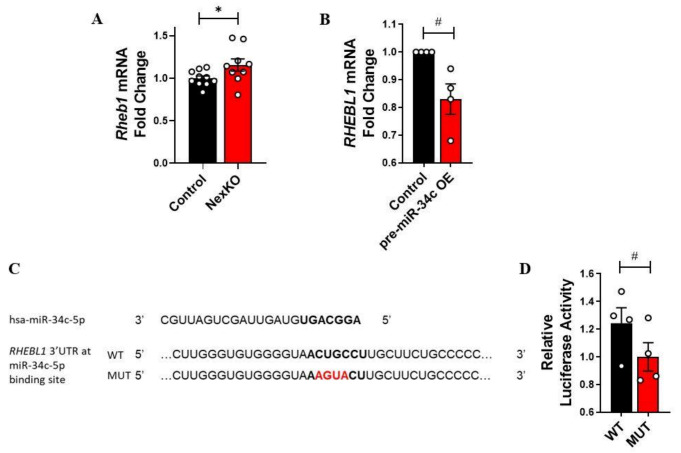
miR-34c-5p does not directly regulate *RHEBL1* mRNA: (**A**) Significantly increased expression levels of *Rheb1* mRNA in the cortices of P30 NexKO mice compared to controls. (**B**) Reduced levels of *RHEBL1* mRNA 72 h post-transfection of hsa-pre-miR-34c in SH-SY5Y cells, compared to controls. (**C**) Sequences of the mature hsa-miR-34c-5p and the Renilla/firefly luciferase psiCHECK2 constructs (WT and MUT) under the regulation of *RHEBL1* 3’UTR, around the hsa-miR-34 binding site; miR-mRNA binding sites are shown in bold; mutated nucleotides are in red. (**D**) Reduced luciferase activity levels of the mutated *RHEBL1* 3’UTR luciferase construct, 72 h post-transfection of hsa-pre-miR-34c in HEK-293T cells, compared to the activity levels of the WT luciferase construct. Data are shown as the mean ± SEM. * *p* < 0.05, # *p* = 0.08; (**A**,**B**) two-tailed *t*-test (**B**) with Welch’s correction; (**D**) one-tailed *t*-test. (**A**) *n* = 10 control; *n* = 9 NexKO; (**B**) *n* = 6 per group; (**D**) *n* = 4 per group. OE: overexpression.

Further bioinformatics analysis revealed several mRNAs of interest that are potential targets of miRNAs of the miR-34 cluster, including mRNAs that are related to forebrain development, gliogenesis, axonogenesis, and neuron migration [100,101,102,103,104,105,106]—all of which were found to be affected in NexKO mice compared to controls [56]. Thus, we hypothesized that the observed downregulation of mmu-miR-34b-5p and mmu-miR-34c-5p in the cortices of P30 NexKO mice compared to controls (Figure 2C,G) would lead to increased expression of suspected targeted mRNAs. To assess this hypothesis, we focused on two miR-34 presumed targets [100,107] that are highly involved in brain development: protein tyrosine phosphatase U (*Ptpru*) [108], and Ras homolog enriched in brain 1 *(Rheb1)* [109,110]. Indeed, RT-qPCR of cortical mRNA validated the expected increases in the expression of *Ptpru* (Figure 3A) and *Rheb1* (Figure 4A).

### 3.4. PTPRU Is Directly Regulated by hsa-miR-34c-5p

*Ptpru* (also known as *Ptpro, PtprΨ*, and *Pcp-2*), which was found to be significantly upregulated in the cortices of P30 NexKO mice compared to controls (Figure 3A), is a member of the R2B subfamily belonging to the ubiquitous protein tyrosine phosphatases (PTPs) family [111,112,113]. PTPs influence essential cellular pathways such as metabolism, differentiation, cell adhesion, cell growth, and the cell cycle [111,114]. Several studies have identified PTPs as crucial components in the development of the nervous system, taking part in neurogenesis, axonogenesis, and the formation and maintenance of neural circuits [108,115]. *Ptpru* was found to be related to neuronal development by protein unfolding [108,111,112,113,116], expressed in many tissues throughout development [111,117,118], and predicted to be regulated by transcription factors that are crucial for the development of the nervous system [108].

Interestingly*, Ptpru* contains a predicted miR-34b/c-binding site on its 3’UTR [107]. Therefore, we hypothesized that the higher *Ptpru* mRNA expression levels in P30 NexKO cortices compared to controls were the result of the reduced expression levels of miR-34b/c-5p, resulting in a reduced inhibitory effect on the expression level of *Ptpru* mRNAs.

To explore the interplay between miR-34b/c-5p and *Ptpru* expression levels, we measured *PTPRU* mRNA expression levels in a human neuronal cell line (SH-SY5Y) following transfection of a plasmid expressing the human (hsa-) pre-miR-34c. SH-SY5Y cells were transfected with either hsa-pre-miR-34c miRVec or an empty control, and miR-34c-5p (Figure 3B) and *PTPRU* mRNA expression levels (Figure 3C) were measured via RT-qPCR. As expected, *PTPRU* mRNA expression levels were significantly lowered in cells transfected with the hsa-pre-miR-34c construct compared to cells transfected with an empty control plasmid, 72 h post-transfection (Figure 3B,C). These results indicate that overexpression of hsa-miR-34c-5p in neuronal cells is correlated with a decrease in the expression level of its putative target, *PTPRU* mRNA.

To examine the direct binding and regulation of hsa-miR-34c-5p on *PTPRU* mRNA expression levels, we utilized the firefly/Renilla luciferase reporter assay and constructed a plasmid that expresses the luciferase mRNA under the regulation of *PTPRU*’s 3’UTR (referred to herein as WT; Figure 3D). As a negative control, we mutated four nucleotides of the hsa-miR-34c-5p seed region on the 3’UTR (referred to herein as MUT; Figure 3D), thus eliminating the miRNA’s capability of binding to the mutated *PTPRU* 3’UTR. HEK-293T cells were transfected with either WT or MUT plasmids, with and without the hsa-pre-miR-34c plasmid. Luciferase activity was quantified 72 h post-transfection. In concordance with our hypothesis, in cells overexpressing hsa-miR-34c-5p, luciferase activity under the WT *PTPRU* 3’UTR was significantly reduced to 75% compared to the normal 100% activity of the mutated 3’UTR (Figure 3E). These results suggest that hsa-miR-34c-5p directly regulates *PTPRU* expression levels through binding to its specific binding site—the 3’UTR of *PTPRU*.

### 3.5. RAS Homolog Enriched in Brain-Like Protein 1 (RHEBL1) Is Not Directly Regulated by hsa-mir-34c-5p

As in the case of *Ptpru*, the expression levels of *Rheb1* were also found to be significantly upregulated in the cortices of P30 NexKO mice compared to controls (Figure 4A). *Rheb1* is a highly conserved gene that is part of the Ras superfamily of small GTPases, which have been shown to be involved in cell growth, differentiation, and proliferation processes [119,120]. *Rheb1* plays a critical role in the differentiation of OL precursor cells (OPCs) into mature, myelinating OLs (mOLs) [114,115,121], but is not necessary for mOLs’ survival or myelin generation and maintenance [110]. Specifically, Rheb1 regulates mTORC1 (mammalian target of rapamycin complex 1) activity by binding to mTOR (mammalian target of rapamycin)—a principal component of mTORC1 [109,120]. mTORC1 activity has been linked to the differentiation process of neural progenitor cells, thus promoting the differentiation of OPCs into mOLs and influencing myelin formation [109,110].

Considering that *Rheb1* has been documented as a positive regulator of OL development and, consequently, myelin formation [109,110], its relatively high levels of expression in the cortices of the myelin-faulted P30 NexKO mice [56], as compared to controls, was surprising. However, since *Rheb1* contains a miR-34c-5p binding site on its 3’UTR, we hypothesized that *Rheb1* expression levels were higher in the cortices of mutant mice compared to controls due to the decrease in miR-34c-5p expression levels in NexKO mice compared to controls. Overexpression of hsa-miR-34c-5p in SH-SY5Y cells (Figure 3B) was correlated with a decrease in *RHEBL1* mRNA expression level 72 h post-transfection (Figure 4B), thus hinting at a possible regulation mechanism. *RHEBL1* is the human paralogue of the murine *Rheb1* that contains a miR-34 binding site on its 3’UTR [107].

However, luciferase reporter assays revealed an increase, rather than a decrease, in luciferase activity when regulated under the WT 3’UTR, relative to the mutated controls (Figure 4C,D). These results suggest that although hsa-miR-34c-5p and *RHEBL1* (or *Rheb1*) have an inverse expression pattern, hsa-miR-34c-5p does not bind *RHEBL1*’s 3’UTR, and the correlation we observed may have been orchestrated in a different manner.

### 3.6. Doublecortin (Dcx)—A Target of miR-34—Was Differentially Expressed in the Cortices of NexKO Mice Compared to Controls and Across Development

Based on our findings of altered WM (Figure 1A,B) and expression levels of miRNAs (Figure 2A,B) involved with biological pathways related to brain development and regulation of cell growth and morphogenesis, we sought to identify these alterations at the molecular level as well. For this purpose, we chose to characterize Dcx properties throughout the mice’s cortical development, since *Dcx* is an accepted molecular indicator of neurogenesis [117,122] and a validated target of the miR-34 cluster [95,96]. Specifically, Dcx has been found to be involved in the regulation of neuronal proliferation, differentiation, and migration, as well as neurite outgrowth and organization [117,118,122,123,124]. *Dcx* encodes a microtubule-associated protein (MAP) that is primarily expressed during neurogenesis and encourages microtubule polymerization [125,126,127]. In mice, *Dcx* expression during embryonic development is ubiquitous in the CNS, and in adulthood it is mainly restricted to neurogenic regions (i.e., the subventricular zone and hippocampus) [117,121]. Nevertheless, *Dcx* expression was also observed in several neocortical areas (e.g., the piriform cortex, cingulate cortex, and entorhinal cortex) of adult mice and rats, although to a lesser degree [117,128].

*Dcx* was previously shown to be regulated through miRNA-RISC inhibition, specifically via direct binding of miR-34a-5p [95] and miR-34c-5p [96] to their binding sites on *Dcx*’s 3’UTR. *Dcx* downregulation through miR-34 binding led to disruptions in neuronal growth and migration, as well as in the cortical morphology of the developing cortices of neonatal rats [95] and porcine embryos [96]. These findings, along with the abnormal brain development in NexKO mice and WS patients compared to controls, prompted us to study Dcx in NexKO mice and their controls.

**Figure 5:**
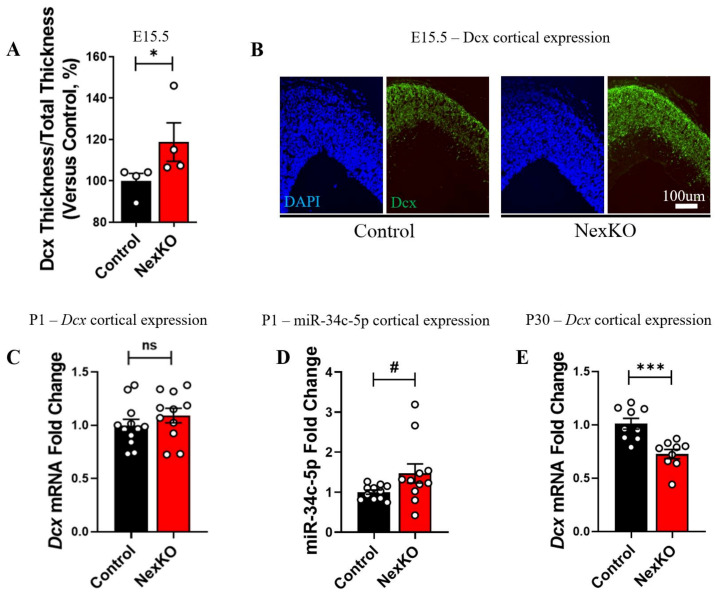
Alterations in Dcx expression levels in the cortices of NexKO embryos, neonates, and young adults compared to age-matched controls: (**A**) Dcx expression in the cortices of E15.5 embryos demonstrate a higher percentage of neuronal layers in the cortices of NexKO embryos compared to controls, as measured by the higher ratio of the thickness of Dcx-positive neuronal layers over the total cortical thickness. (**B**) Representative confocal images showing staining of cell nuclei (DAPI, in blue) and Dcx (green) in the cortices of E15.5 control and NexKO embryos. (**C**) Comparable *Dcx* mRNA levels in P1 NexKO mice compared to controls. (**D**) A trend towards an increase in miR-34c-5p levels in the cortices of P1 NexKO mice compared to controls. (**E**) Significantly decreased expression levels of *Dcx* mRNA in the cortices of P30 NexKO mice compared to controls. Data are shown as the mean ± SEM. * *p* < 0.05, *** *p* < 0.005, # *p* = 0.08 (**A**) two-tailed Mann–Whitney test, (**C**–**E**) two-tailed *t*-test with Welch’s correction. (**A**) *n* = 4 per group; (**C**) *n* = 12 control; *n* = 11 NexKO; (**D**) *n* = 11 per group; (**E**) *n* = 9 per group. ns: non-significant.

Therefore, in order to study whether Dcx properties in the brains of NexKO mice are altered throughout the development of the CNS compared to controls, we characterized its properties in embryonic, early-postnatal, and adult mice. Interestingly, NexKO E15.5 embryos showed significantly greater cortical thickness compared to controls, measured via staining of Dcx (Figure 5A,B). These results are in accordance with Dcx’s developmental role in the CNS and the altered differentiation and development of the CNS in NexKO mice compared to controls [56].

To further examine *Dcx*’s role throughout the development of the CNS, as well as its interplay with miR-34c-5p, we measured *Dcx* mRNA and miR-34c-5p expression levels in the cortices of P1 mice. *Dcx* expression levels were comparable at P1 between the cortices of P1 NexKO mice and controls (Figure 5C). RT-qPCR analysis showed a non-significant trend towards an increase in miR-34c-5p expression levels in P1 NexKO cortices compared to controls (Figure 5D). Examining *Dcx* later in development, we found significantly decreased expression levels of *Dcx* in the cortices of P30 NexKO mice compared to controls (Figure 5E). This suggests that although miR-34b/c-5p expression levels in P30 NexKO cortices were decreased relative to controls (Figure 2C,G), *Dcx* mRNA levels were significantly decreased, rather than increased—possibly via a different regulation process. Taken together, *Dcx* and miR-34c-5p expression patterns in the cortices of NexKO mice compared to controls during development suggest a unique developmental association between miR-34c-5p and *Dcx*. Further experiments should be conducted in order to deepen the understanding of the interaction of miR-34c-5p and *Dcx* throughout cortical development in NexKO mice.

## 4. Discussion

The selective deletion of *Gtf2i* from excitatory neurons of the forebrain resulted in WS-relevant abnormalities, including neuroanatomical defects and increased sociability and anxiety [56]. Moreover, NexKO brains exhibited dramatic disruptions in the expression of myelin- and differentiation-related gene transcripts, axon myelination properties, and neuronal function [56].

Utilizing DTI—a diffusion MRI framework that is sensitive enough to detect alterations in tissue microstructures [125]—we characterized significant changes in diffusivity indices in multiple affected brain regions. Our analysis focused on the corpus callosum (CC) and the limbic outputs such as the fimbria/fornix fibers and the stria terminalis, due to previous evidence we discovered showing myelin deficits in the CC, and due to the roles of the fimbria/fornix and the stria terminalis in mediating behavior. WM alterations such as those we found may lead to aberrant regulation and synchronization of the signal transduction in neural circuits [129], which is critical for proper brain activity and behavior [126].

The dramatic transcriptional alterations in multiple mRNAs related to brain-development-related pathways [56] led us to consider the possibility of the existence of potential miRNAs that may act as “master regulators” of these mRNAs. To examine this possibility, we utilized next-generation sequencing (NGS) techniques, which revealed considerable alterations in the expression levels of several miRNAs of interest in NexKO mice compared to controls. Among these were several miRNAs that were previously linked to brain development, such as miR-10b-5p [87,89], miR-145a-5p [90], miR-221-5p [91], miR-29c-5p [92], miR-186-5p [93], miR-34b-5p [94], and miR-34c-5p [94].

Of the differentially expressed miRNAs, we chose to single out two miRNAs that have been extensively studied in the context of brain development [97,100,101]: mmu-miR-34b-5p and mmu-miR-34c-5p (herein referred to as miR-34b/c). These miRNAs have validated or presumed targets [107,130], which we found to be differentially expressed in the cortices of the NexKO mice compared to controls [56]. These miRNAs are transcribed under the same promoter on chromosome 9 [94,96,127,131,132,133,134,135], and belong to the evolutionarily conserved miR-34 cluster, which is known to be involved in cell differentiation and migration [94]—processes that were disrupted in the cortices of P30 NexKO mice compared to controls [56]. Moreover, miR-34b/c seem to be primarily expressed in neocortical neurons [136,137,138], suggesting that the observed downregulation occurred mainly in neurons in the cortices of NexKO mice compared to controls.

Numerous studies have shown the critical role of the miR-34 family in neuronal differentiation [97,100,101,139]. For example, depletion of miR-34c-5p in murine fetuses at embryonic day 14.5 (E14.5) induced an increase in cell proliferation at E17.5, while an increase in miR-34c-5p expression levels induced the opposite effect [96]. Specifically, miR-34b/c were shown to be involved in the Wnt signaling pathway [99]—a canonical pathway involved in cell proliferation, differentiation, and migration [99,140]—and to be linked to *P53*, a tumor-suppressor gene that encodes the stress-activated transcription factor TP53 [94,141,142,143].

Nevertheless, although miR-34b/c involvement in brain development is rather well founded [97,100,101,139], it cannot be concluded that it is the sole regulator of brain development. For instance, Wu et al. (2014) showed that double KO of the miR-34 cluster together with the miR-449 cluster (which share the same functional targets) causes a severe disruption in the brain development of mutated mice, as well as a reduced brain volume [98]—possibly through aberration of the mitotic spindle orientation in cortical progenitors—which results in delayed neuronal differentiation [144]. However, a single KO of the miR-34 cluster led to an increase in miR-449 cluster expression, which may have compensated for the lack of miR-34 and prevented the impairment in brain development [98].

The current study showed that two speculated mRNA targets of the miR-34 cluster—*Ptpru* and *Rheb1*—were significantly overexpressed in the cortices of P30 NexKO mice compared to controls. Luciferase reporter assays performed in human cell lines revealed that *PTPRU* was indeed targeted by hsa-miR-34c-5p through direct binding of the miR’s binding site to *PTPRU*’s 3’UTR.

*Ptpru*—a member of the R2B PTP family—regulates tyrosine phosphorylation and cadherin-based cell adhesion, and was found to be involved in CNS development [108,112]. *Ptpru* regulates neurite extension by inactivating β-catenin—a player in the Wnt signaling pathway [111,145,146]. The Wnt/β-catenin signaling pathway is a prominent regulator of differentiation of OPCs into mOLs [139,147,148]. Specifically, Ortega et al. (2013) showed that activation of the canonical Wnt signaling pathway induced oligodendrogenesis, while activation of the non-canonical Wnt pathway reduced its levels [139]. Thus, it is possible that the upregulated *Ptpru* levels observed in the cortices of NexKO mice compared to controls led to inhibition of β-catenin [149] and, thus, the canonical Wnt signaling pathway [148], which may have led to the faulted differentiation of OPCs to mOLs [139,148] that we observed in the cortices of NexKO mice [56]. As previously mentioned, miR-34b/c were also associated with the Wnt signaling pathway [99]. Our novel discovery that hsa-miR-34c-5p directly downregulates *PTPRU* mRNA expression illuminates a previously unknown element in the Wnt signaling cascade and its regulation. Nevertheless, miR-34b/c were previously found to directly downregulate *Wnt1* [99], and since *Ptpru* expression was inversely correlated with *Wnt1* in the midbrains of chicks [150], this implies that there may be other players involved. Another explanation is that the miRNA’s regulatory function is activated only in specific timepoints and cell types, as happens with other types of miRNAs [67].

Rheb1 is a direct activator of mTOR1, and was found to be critical for the differentiation of OPCs into mOLs [110]. The present study revealed significantly higher expression level of *Rheb1* in the cortices of P30 NexKO mice compared to controls. In addition, hsa-miR-34c-5p overexpression in human neuronal cell lines resulted in a decrease in the expression level of *RHEBL1*. However, *RHEBL1* was not found to be directly regulated by hsa-miR-34c-5p via luciferase reporter assay in HEK-293 cells. *Rheb1* expression levels are dependent upon the developmental stage and cellular context [151]. This variability in *Rheb1* expression can explain why we measured different expression characteristics of *Rheb1* (or *RHEBL1*) in CNS cells (murine cortex and neuronal cell lines) compared to embryonic kidney cells (HEK-293T). This explanation is supported when considering that miRNAs are also susceptible to such contextual changes [67]. Thus, it is possible that in the neuronal context, miR-34c-5p targeting of *Rheb1* is favored over other targets, but in HEK-293T cells this is not the case.

Nevertheless, a distinct consideration should be given to the caveats existing in the translation of scientific findings from animal to human contexts [152,153]. In the present study, such an obstacle occurred in the attempt to explore a mechanism that was hypothesized in the murine context but orchestrated in a human one—specifically, hypothesizing that miR-34b/c target the murine *Rheb1*, but exploring them in the human context with *RHEBL1*’s 3’UTR. Although an orthologue for *Rheb1* exists in humans, and is transcribed from chromosome 7, it does not contain a binding site for miR-34b/c on its 3’UTR [145]. However, *RHEBL1*, which is transcribed from chromosome 12, does contain such a binding site on its 3’UTR [145] and, therefore, was what we used in the human-context experiments. At any rate, it is possible that miR-34b/c exert their inhibitory function on the murine *Rheb1*, but not on the human *RHEBL1*.

Additional molecular evidence for altered brain development in NexKO mice compared to controls is Dcx expression in the cortex throughout development. Dcx is a microtubule-binding protein known to be highly expressed in differentiating and migrating neurons [121]. Dcx aids in axonal outgrowth by promoting microtubule polymerization and stability [121,122]. The current study found *Dcx*—a known target of the miR-34 family [95,96]—to be significantly downregulated in P30 NexKO cortices compared to controls, despite the reduced expression of its known downregulators miR-34b/c. In addition, *Dcx* mRNA expression levels in the cortices of P1 NexKO pups were similar to those observed in control pups. Interestingly, our findings of increased Dcx protein expression levels in the cortices of E15.5 NexKO embryos compared to controls may hint at a reduced expression of miR-34c-5p, since it was previously shown that depletion of miR-34c-5p at E14.5 induced an increase in neuronal differentiation at E17.5 [96]. Considering the involvement of *Dcx* in the developing cortex [121], it is of no surprise that *Dcx* shows developmentally altered expression levels in the murine cortex.

All the same, recent reports show that Dcx plays additional roles besides microtubule organization in developing neurons [132]; for instance, Klempin et al. (2011) found *Dcx* to be expressed in post-mitotic neurons in the murine piriform cortex, outside the commonly accepted neurogenic regions [128]. The murine piriform cortex is known to be involved in the processing of olfactory information [128]. Therefore, Klempin et al. suggested that the *Dcx*-expressing neurons located there are involved in synaptic plasticity and adaptation to environmental changes [128]. Hence, it is possible that the reduction in *Dcx* expression levels observed in the NexKO cortices in comparison with controls is associated with a deficit in plasticity-related neurons, which results in aberrated adjustment to novel environments. This conjecture is consistent with the elevated anxiety levels we previously observed in NexKO mice compared to controls [56]. Moreover, it was recently shown that *Dcx*-enriched neuronal precursor cells are able to differentiate into mOLs in the murine hippocampus when demyelination is induced [146]. As was shown here and in previous studies, myelin and OL differentiation properties are aberrated in the brains of NexKO mice compared to controls [34,56]. Hence, it is possible that this proposed myelination-repair mechanism [146] is faulted in the brains of NexKO mice, perhaps due to the alterations in miR-34b/c and *Dcx* expression levels, and their impact on neurogenesis. Furthermore, a recent study revealed that not only differentiating or migrating neurons, but also OPCs express *Dcx*, and that *Dcx* expression is downregulated in mOLs [154]. Therefore, the reduced expression of *Dcx* in the P30 NexKO cortex compared to controls may originate from a greater OPC-to-mOL ratio in the NexKO mice compared to controls [57].

The formulation of a novel murine model for the hypersociability phenotype observed in WS has provided us with the opportunity to examine social behavior from a new perspective. Recognition of the vast effect *Gtf2i* has on brain development, cell differentiation, cortical development, and myelination enables the investigation of these properties in the context of social behavior. Our study suggests that the developmental aberrations induced by the selective deletion of *Gtf2i* from excitatory neurons of the forebrain [56] may be also mediated by alterations in miR-34c-5p expression levels and its regulation of neurodevelopment-related targets, starting at the embryonic stage and observed at P30. Nevertheless, further research should be carried out in order to deepen the understanding of miR-34 involvement in the development of *Gtf2i*-deficient brain. Singling out miRNAs that may regulate neurodevelopmental processes promotes the possibility of novel therapies for WS specifically, or for neurodevelopmental disorders in general.

## Figures and Tables

**Table 1:** PCR primers for genotyping of NexKO or control mice.

Name of Primer	Sequence
Nex-Cre fwd	GAGTCCTGGAATCAGTCTTTTTC
Nex-Cre rev	AGAATGTGGAGTAGGGTGAC
Nex-Cre KO	CCGCATAACCAGTGAAACAG

**Table 2:** PCR primers for sex determination of mice.

Name of Primer	Sequence
Rmb31x/y fwd	CACCTTAAGAACAAGCCAATACA
Rmb31x/y rev	GGCTTGTCCTGAAAACATTTGG

**Table 3:** SYBR Green RT-PCR primers for mRNA quantification: Mus musculus.

Origin	Name of Primers	Sequence
*Gapdh*	Gapdh fwd	GCCTTCCGTGTTCCTACC
	Gapdh rev	CCTCAGTGTAGCCCAAGATG
*Ptpru*	Ptpru fwd	GTGGACAAGTGGCAGGCAGA
	Ptpru rev	CAGGCTGTGACAGCGGATCA
*Rheb1*	Rheb1 fwd	TTGTTGATTCCTACGATCCAACCA
	Rheb1 rev	CCGCTGTGTCTACAAGCTGAAGATG
*Dcx*	Dcx fwd	CATCACAGAAGCGATCAAACTGGA
	Dcx rev	CAGGACCACAAGCAATGAACACA

**Table 4:** SYBR Green RT-PCR primers for mRNA quantification: Homo sapiens.

Origin	Name of Primers	Sequence
*Β-ACTIN*	Has-β-actin fwd	CCTGGACTTCGAGCAAGAGATGG
	Has-β-actin rev	TGGAGTTGAAGGTAGTTTCGTGGATG
*PTPRU*	Has-ptpru fwd	ACCTGTACCGCTGTGTGTCCCA
	Has-ptpru rev	GGAGTTGGTGTTGAGCTGGATGA
*RHEBL1*	Has-rhebl1 fwd	GATAGTGACTCTTGGCAAAGATGAGTT
	Has-rhebl1 rev	TGGACCCCAATGATGAATGAA

**Table 5:** TaqMan RT-PCR probe and primer assays for miRNA quantification.

miRNA	Mature miRNA Sequence	Thermo Fisher Scientific Assay ID
U6 snRNA	GTGCTCGCTTCGGCAGCACATATACTAAAATTGGAACGATACAGAGAAGATTAGCATGGCCCCTGCGCAAGGATGACACGCAAATTCGTGAAGCGTTCCATATTTT	001973
hsa-miR-34c-5p	AGGCAGUGUAGUUAGCUGAUUGC	478052_mir

**Table 6:** hsa-PTPRU primer sequences for fragment amplification, cloning, and mutagenesis.

Purpose	Name of Primers	Sequence *
WT	XhoI-PTPRU 3’UTR fwd	TACATCG***CTCGAG***TTGGCAGGGATGAGTGAGGC
	NotI-PTPRU 3’UTR rev	TA***GCGGCCGC***CGAGGTGACTTCATTCTGCAACA
Mutant	mut-PTPRU 3’UTR fwd	CAAAATATCTCAGGGGCTGCAGG***GTTACTGT***GG GAGGAGGGCGCTGCAGTTCCCC
	mut-PTPRU 3’UTR rev	GGGGAACTGCAGCGCCCTCCTCCC***ACAGTAA***CC CTGCAGCCCCTGAGATATTTTG

* XhoI or NotI restriction sites and mutated nucleotides are marked in bold and italics.

**Table 7:** hsa-RHEBL1 primer sequences for fragment amplification, cloning, and mutagenesis.

Purpose	Name of Primers	Sequence *
WT	XhoI-RHEBL1 3’UTR fwd	TACATCG***CTCGAG***CCATCTCATGTGAGCCCTTGG
	NotI-RHEBL1 3’UTR rev	TA***GCGGCCGC***GCCAGTGTCCATGAGAGGTCCT
Mutant	mut-RHEBL1 3’UTR fwd	CCGGGGGCAGAAGCA***AGTACT***TTACCCCACACC CAAGGGC
	mut-RHEBL1 3’UTR rev	GCCCTTGGGTGTGGGGTAA***AGTA***CTTGCTTCTGC CCCCGG

* XhoI or NotI restriction sites and mutated sites are marked in bold and italics.

**Table 8:** pre-miR-34c primer sequences for fragment amplification and cloning.

Name of Primers	Sequence *
BamHI-pre-miR- 34c fwd	TGC***GGATC***CCTCAACCAATGAATTGCCTGCC
pre-miR- 34c rev	CCACGCACATTGATGATGCACA

* BamHI restriction site is marked in bold and italics.
